# Targeting PI3K signaling in Lung Cancer: advances, challenges and therapeutic opportunities

**DOI:** 10.1186/s12967-025-06144-8

**Published:** 2025-02-14

**Authors:** Bitian Zhang, Ping-Chung Leung, William Chi-Shing Cho, Chun-Kwok Wong, Dongjie Wang

**Affiliations:** 1 Institute of Chinese Medicine, State Key Laboratory of Research on Bioactivities and Clinical Applications of Medicinal Plants, The Chinese University of Hong Kong, Hong Kong, China; 2 Department of Clinical Oncology, Queen Elizabeth Hospital, Hong Kong, China; 3 Department of Chemical Pathology, Prince of Wales Hospital, The Chinese University of Hong Kong, Hong Kong, China; 4 Li Dak Sum Yip Yio Chin R & D Centre for Chinese Medicine, The Chinese University of Hong Kong, Hong Kong, China; 5 Shenzhen Research Institute, The Chinese University of Hong Kong, Shenzhen, China

## Abstract

Lung cancer remains the leading cause of cancer-related mortality globally, necessitating the continual exploration of novel therapeutic targets. The phosphoinositide 3-kinase (PI3K) signaling pathway plays a pivotal role in oncogenic processes, including cell growth, survival, metabolism and immune modulation. This comprehensive review delineates the distinct roles of PI3K subtypes—PI3Kα, PI3Kβ, PI3Kγ and PI3Kδ—in lung cancer pathogenesis and progression. We evaluate the current landscape of PI3K inhibitors, transitioning from non-selective early-generation compounds to isoform-specific agents, highlighting their clinical efficacy, resistance mechanisms and potential combination strategies. Furthermore, the intricate interplay between PI3K signaling and the tumor immune microenvironment is explored, elucidating how PI3K modulation can enhance immunotherapeutic responses. Metabolic reprogramming driven by PI3K signaling is also dissected, revealing vulnerabilities that can be therapeutically exploited. Despite promising advancements, challenges such as therapeutic resistance and adverse effects underscore the need for personalized medicine approaches and the development of next-generation inhibitors. This review underscores the multifaceted role of PI3K in lung cancer and advocates for integrated strategies to harness its full therapeutic potential, paving the way for improved patient outcomes.

**Supplementary Information:**

The online version contains supplementary material available at https://doi.org/10.1186/s12967-025-06144-8.

## Introduction

Lung cancer, encompassing non-small cell lung cancer (NSCLC) and small cell lung cancer (SCLC), accounts for the highest number of cancer-associated deaths worldwide [1], driven by factors such as late-stage diagnosis, metastatic spread, and therapeutic resistance. Despite significant advancements in targeted therapies and immunotherapies, the prognosis for lung cancer patients remains poor, with a pressing need for novel therapeutic targets and strategies [2]. Among the myriad molecular pathways implicated in lung oncogenesis, the phosphoinositide 3-kinase (PI3K) signaling axis has emerged as a central player, orchestrating critical cellular processes that underpin tumor growth, survival, metabolism, and immune evasion [3].

The PI3K family comprises seven isoforms categorized into three classes [4], with Class I PI3Ks—PI3Kα, PI3Kβ, PI3Kγ, and PI3Kδ—being particularly relevant in the context of lung cancer. These isoforms exhibit distinct regulatory mechanisms and cellular functions, contributing to the heterogeneity and adaptability of lung tumors. PI3Kα, for instance, is frequently activated through genetic alterations such as PIK3CA mutations, driving processes like epithelial-to-mesenchymal transition (EMT) and metastasis [5]. PI3Kβ has been implicated in therapeutic resistance, especially in tumors deficient in the tumor suppressor PTEN [6], while PI3Kγ and PI3Kδ are integral to modulating the immune landscape within the tumor microenvironment (TME) [7, 8].

Targeting the PI3K pathway offers a promising therapeutic avenue, with the development of PI3K inhibitors progressing from broad-spectrum agents to more selective compounds that aim to maximize efficacy while minimizing off-target effects. These inhibitors not only disrupt oncogenic signaling but also influence the immune milieu, potentially synergizing with immunotherapies to enhance anti-tumor responses [9]. Moreover, the PI3K/AKT/mTOR axis is a key regulator of metabolic reprogramming in cancer cells [10], facilitating the adaptation to nutrient-deprived and hypoxic conditions typical of the TME.

However, the clinical application of PI3K inhibitors is challenged by issues such as drug resistance, adverse immunological effects, and the intrinsic complexity of the PI3K signaling network [11]. Understanding the specific roles of PI3K isoforms in lung cancer biology, elucidating resistance mechanisms, and devising combination therapies are critical for overcoming these hurdles. Additionally, the interplay between PI3K signaling and metabolic pathways offers opportunities to exploit metabolic vulnerabilities in tumor cells.

This review aims to provide a comprehensive overview of the current understanding of PI3K signaling in lung cancer, encompassing the distinct functions of PI3K subtypes, the therapeutic landscape of PI3K inhibitors, their impact on immune modulation within the TME, and their role in metabolic control. By integrating insights and analysis from preclinical and clinical studies, we highlight the potential of PI3K-targeted therapies to transform lung cancer treatment paradigms and identify strategies to enhance their clinical efficacy. Ultimately, this synthesis of current knowledge seeks to shed light for future research directions and facilitate the translation of PI3K-targeted approaches into effective clinical interventions for lung cancer patients.

### PI3K subtypes in lung cancer

The PI3K family consists of seven isoforms categorized into three classes (I, II, and III) [12]. In the context of lung cancer, Class I PI3Ks are the most relevant, comprising four isoforms: PI3Kα, PI3Kβ, PI3Kγ, and PI3Kδ [13, 14]. Each subtype exhibits distinct regulatory domains, substrate specificities, and cellular localizations, contributing to diverse cellular processes.

PI3Kα is predominantly involved in regulating cell growth, survival, and angiogenesis, and is also frequently activated in lung adenocarcinomas through genetic alterations such as PIK3CA mutations and amplifications [15]. PI3Kα activation is a critical event in the epithelial-to-mesenchymal transition (EMT), a process that enhances metastatic potential by promoting cell motility and invasiveness. For instance, studies have shown that PIK3CA mutations correlate with increased EMT marker expression and higher rates of metastasis in NSCLC [16]. PI3Kβ plays a crucial role in cell cycle progression and survival. PI3Kβ is implicated in therapeutic resistance, particularly in tumors lacking PTEN, where it facilitates Akt activation independent of PI3Kα [6]. While PI3Kγ and PI3Kδ are primarily associated with immune cell function, their roles in lung cancer extend beyond immune modulation. PI3Kγ activation promotes the accumulation of immunosuppressive myeloid cells in the TME [17].

### Aberrant activation of PI3K/AKT/mTOR pathway in lung cancer

The PI3K/AKT/mTOR signaling axis is fundamental to cellular processes such as growth, proliferation, survival, and metabolism. In lung cancer, dysregulation of this pathway is a common event, rendering it a critical target for therapeutic intervention [18]. Activation of the PI3K/AKT/mTOR pathway is a hallmark of NSCLC, correlating with poor prognosis, increased tumor aggressiveness, and resistance to various therapies [19]. The mechanisms underlying pathway activation in lung cancer are multifaceted, including mutations in PI3K genes, aberrant expression of PTEN, overexpression of downstream effectors and activation of upstream kinases [20].

Mutations in the PIK3CA gene are among the most common genetic alterations leading to PI3K pathway activation in lung cancer. These mutations, particularly in the helical and kinase domains, result in constitutive PI3K activity, driving oncogenic signaling independent of external growth factors [21]. For example, the H1047R mutation in PIK3CA enhances the lipid kinase activity of PI3Kα, promoting increased PIP3 production and sustained AKT activation [22].

PTEN loss is another critical factor contributing to PI3K pathway activation. PTEN functions as a lipid phosphatase, counteracting PI3K by dephosphorylating PIP3 to PIP2. In NSCLC, PTEN loss occurs through various mechanisms, including genetic deletions, promoter methylation, and post-translational modifications [23]. The absence of functional PTEN leads to persistent AKT activation, and enhanced cancer cell survival and proliferation with the overexpression of PD-L1 in TME [24].

Elevated expression levels of downstream effectors, such as AKT and mTOR, further potentiate PI3K pathway signaling. In lung adenocarcinoma, increased AKT expression correlates with enhanced tumor aggressiveness and poor clinical outcomes [25]. Similarly, overactivation of mTORC1 and mTORC2 complexes facilitates robust protein synthesis and metabolic reprogramming, supporting rapid tumor growth [26, 27].

Upstream receptor tyrosine kinases (RTKs) like EGFR and HER2 are frequently overexpressed or mutated in lung cancer, leading to enhanced PI3K pathway activation. For instance, EGFR mutations in NSCLC not only drive increased PI3K signaling but also contribute to resistance against EGFR tyrosine kinase inhibitors (TKIs) through PI3K-mediated survival pathways [28]. Moreover, HER2 amplification has been implicated in the activation of the PI3K/AKT/mTOR axis, promoting tumor proliferation and resistance to chemotherapy [29, 30].

### Effect of PI3K on immunity in lung cancer

PI3K signaling plays a pivotal role in regulating immune responses within the TME of lung cancer. The PI3K/AKT/mTOR axis governs essential cellular processes such as survival, growth, and metabolism, while also influencing immune checkpoint pathways that tumors exploit to evade immune surveillance [31]. Dysregulation of PI3K signaling is a prevalent feature in lung cancer, often correlating with resistance to immune checkpoint inhibitors (ICIs) and presenting significant hurdles to effective immunotherapy.

Activation of the PI3K/AKT/mTOR pathway leads to the upregulation of immune checkpoint ligands, notably programmed death-ligand 1 (PD-L1). Elevated PD-L1 expression on tumor cells facilitates the suppression of CD8^+^ T-cell activity and promotes the expansion of regulatory T cells (Tregs), thereby attenuating anti-tumor immunity and allowing unchecked cancer cell proliferation [32, 33]. Moreover, PI3K signaling contributes to an immunosuppressive TME by enhancing the infiltration and functionality of myeloid-derived suppressor cells (MDSCs) and tumor-associated macrophages (TAMs) [34, 35]. These immunosuppressive cell populations further inhibit effective immune responses, fostering an environment conducive to tumor growth and resistance to immunotherapeutic interventions [36].

The interplay between PI3K signaling and immune checkpoint pathways provides a compelling rationale for combination therapies. PI3K inhibitors disrupt tumor-promoting signaling pathways and enhance immune cell responsiveness to ICIs, resulting in more effective immune-mediated tumor suppression. PI3K inhibition can remodel the TME by reducing the prevalence of MDSCs and TAMs, thereby alleviating immune suppression and fostering a more immunostimulatory milieu. This reconfiguration facilitates the infiltration and functionality of cytotoxic T cells, which are essential for robust antitumor responses. Additionally, PI3K inhibitors directly enhance the proliferation and activity of effector T cells, bolstering their capacity to eliminate tumor cells [37]. Notably, PI3Kδ inhibitors have demonstrated effectiveness in diminishing Treg-mediated suppression, thereby further enhancing immune-mediated tumor control [38].

Despite the promising potential of integrating PI3K inhibitors with ICIs, several challenges must be addressed to optimize this therapeutic strategy. A primary concern is the heightened risk of toxicity, as both PI3K inhibitors and ICIs can induce immune-related adverse events [39, 40]. Determining optimal dosing regimens and sequencing strategies is crucial to maximize therapeutic benefits while minimizing adverse effects. Additionally, the emergence of the resistance to PI3K inhibitors presents a significant hurdle [41], necessitating a deeper understanding of resistance mechanisms to inform the development of more effective combination therapies. Ongoing research aimed at elucidating these mechanisms and refining combination protocols will be essential for translating these strategies into widespread clinical success.

Future directions include the identification of biomarkers to predict response to PI3K inhibition and ICI combination therapies, thereby enabling personalized treatment approaches. Furthermore, exploring the role of different PI3K isoforms in modulating immune responses may uncover novel therapeutic targets and strategies. Advances in nanotechnology and drug delivery systems also hold promise for enhancing the specificity and reducing the toxicity of PI3K inhibitors, thereby improving their therapeutic index when used in combination with ICIs.

### PI3K and metabolic control

Metabolic reprogramming is a hallmark of cancer, enabling tumor cells to adapt to the nutrient-deprived and hypoxic TME. The PI3K/AKT/mTOR pathway is central to this metabolic reprogramming in lung cancer, orchestrating anabolic and catabolic processes to sustain rapid cell growth and survival [42].

Generally, PI3K mediate cancer metabolism in the following manners, including enhancing anabolic metabolism, modulation of metabolic enzymes and further suppression of metabolic stress and autophagy.

PI3K activation promotes anabolic metabolism by increasing glucose uptake and glycolysis, facilitating the synthesis of nucleotides, amino acids, and lipids. In lung cancer cells, PI3K-mediated signaling enhances the expression of glucose transporter 1 (GLUT1), thereby boosting glycolytic flux and providing substrates for biosynthetic pathways [43, 44]. Additionally, PI3K/AKT signaling stimulates lipid synthesis through the activation of sterol regulatory element-binding proteins (SREBPs), supporting membrane biogenesis and energy storage [45]. PI3K inhibition disrupts anabolic metabolism while simultaneously activating catabolic processes [46]. This shift creates a metabolic crisis in lung cancer, impairing their ability to sustain growth and survive under therapeutic stress [47].

Besides, PI3K inhibitors have been shown to downregulate key enzymes and transporters involved in various metabolic pathways. For instance, buparlisib suppresses the expression of hexokinase 2 (HK2) and pyruvate kinase M2 (PKM2) [48, 49], critical glycolytic enzymes, thereby hindering glycolytic flux and energy production in cancer cells. Moreover, PI3K inhibition impacts lipid metabolism by reducing the expression of fatty acid synthase (FASN), limiting lipid biosynthesis necessary for membrane formation and energy storage [50]. These alterations underscore the pivotal role of PI3K in maintaining metabolic homeostasis in lung cancer cells.

Furthermore, PI3K inhibitors induce metabolic stress in lung cancer cells by limiting nutrient availability and energy production. This stress can activate autophagy, a catabolic process that recycles cellular components to maintain energy homeostasis [51, 52]. While autophagy initially serves as a survival mechanism, prolonged inhibition can lead to cell death, presenting a therapeutic opportunity to exploit further metabolic vulnerabilities in cancer cells.

Combining PI3K inhibitors with metabolic modulators can enhance therapeutic efficacy by concurrently targeting multiple metabolic pathways. Several combinations have demonstrated synergistic antitumor effects in preclinical models of lung cancer. Pan-PI3K inhibitor buparlisib combined with AMPK activator metformin was found to induce lung cancer cell apoptosis via Akt/FoxO3a/Puma pathway [53]. Dual inhibition on PI3K and mTOR could not only directly suppress lung caner progression, but also enhance the efficacy of chemotherapy or targeted therapy in lung cancer treatment [54]. While the synergistic inhibition of tumor growth by PI3K/mTOR inhibitor may due to simultaneously disrupts anabolic processes.

In brief summary, the PI3K/AKT/mTOR pathway plays a crucial role in regulating metabolic processes that support the growth and survival of lung cancer cells. PI3K inhibitors disrupt anabolic metabolism, modulate key metabolic enzymes, and induce metabolic stress and autophagy, thereby impairing tumor growth. Furthermore, combining PI3K inhibitors with metabolic modulators, such as AMPK activators or glutaminase inhibitors, can enhance therapeutic efficacy by targeting multiple facets of cancer metabolism. Ongoing clinical trials and preclinical studies continue to explore these combinations, offering promising avenues for improving outcomes in patients with lung cancer.

### PI3K inhibitors in lung cancer therapy

Significant advancements have been made in the investigation of PI3K inhibitors, with a multitude of these therapeutic agents currently undergoing preclinical studies [4, 55–60]. The research endeavors have led to a robust pipeline of PI3K inhibitors that are being rigorously evaluated for their efficacy and safety profiles prior to clinical translation. Apart from that, the U.S. Food and Drug Administration (FDA) had granted approval for a total of 69 small-molecule protein kinase inhibitors up to 2024 that are specifically for cancer therapy. While only 5 PI3K inhibitors have been granted so far, including copanlisib, alpelisib, idelalisib, duvelisib, and umbralisib [61]. The modest count of approved PI3K inhibitors marks a notable milestone in the evolution of targeted oncological treatments. This progress, while commendable, simultaneously reveals the extensive terrain that remains to be explored in the quest for novel PI3K inhibitory agents.

PI3K inhibitors represent a significant advancement in the targeted therapy of lung cancer, effectively disrupting critical signaling pathways involved in tumor growth and survival. Ongoing advancements in inhibitor design, strategic combination therapy approaches, and resistance mitigation are pivotal for maximizing clinical efficacy. Modern PI3K inhibitors are categorized based on their selectivity: PI3Kα, PI3Kβ, PI3Kγ, PI3Kδ inhibitors, and pan-PI3K inhibitors that target multiple isoforms simultaneously. Currently, there are 11 PI3K inhibitors in clinical trial that are specifically used for lung cancer therapy (Table 1).

**Table 1:** Current Clinical Trials Evaluating PI3K Inhibitors in Lung Cancer Therapy

Target	Name	Phase	NCT trial for lung cancer
pan-PI3K inhibitor	PX-866	II	NCT01204099
	XL147/Pilaralisib	I	NCT04895579
	GDC-0941/Pictilisib	II	NCT01493843
	PKI-584/Gedatolisib	II	UMIN000020585
PI3Kα	BYL719/Alpelisib	II	NCT02276027
	GDC-0032/Taselisib	II	NCT02785913
	TAK117/Serabelisib	I	NCT01449370
PI3Kβ	AZD8186	I	NCT01884285
	GSK2636771	I/​IIa	NCT01458067
PI3Kδ	CAL-101/Idelalisib	Ib/II	NCT03257722
	TQ-B3525	II	NCT05284994

### Pan-PI3K inhibitors

The pan-PI3K inhibitors such as Wortmannin and LY294002, were non-selective, targeting multiple PI3K isoforms and subsequently limiting their clinical utility due to significant off-target effects and poor pharmacokinetic profiles [62, 63]. PX-866 is a semi-synthetic derivative of wortmannin, which functions as an orally available and irreversible pan-PI3K inhibitor [64]. Though PX-866 potently inhibit PI3Kα/γ/δ, it demonstrates reduced on-target toxicity compared to wortmannin due to limited inhibition on PI3Kβ [65]. So far, a phase II trial of PX-866 on NSCLC has been conducted, yet the addition of PX-866 to docetaxel failed to improve progression-free survival (PFS), response rate, or overall survival (OS) in patients with advanced and refractory NSCLC [66].

XL147/pilaralisib is a quinoxaline scaffold, reversible ATP-competitive pan-PI3K inhibitor [67]. A phase I clinical trial on pilaralisib has been conducted in patients with advanced solid tumors including squamous cell lung cancer, and the results showed that pilaralisib demonstrated a favorable safety profile and preliminary antitumor activity. Besides, The recommended phase II dose for pilaralisib tablets was calculated as 400 mg once daily based on PK data [68]. GDC-0941/pictilisib is another p*an-PI3K inhibitor for lung cancer therapy that has been conducted for clinical trial. However*,* a phase II study for* pictilisib failed to improve PFS and OS in non-squamous and squamous NSCLC patients (NCT01493843) [69].

PKI-584/gedatolisib is a dual PI3K/mTOR inhibitor [70, 71]. It is reported that gedatolisib significantly suppressed SCLC in preclinical experiments. Further investigation showed that purine metabolites have emerged as critical mediators that contributed to the development of resistance to gedatolisib [72]. However, a phase II clinical trial conducted in Japan has demonstrated that gedatolisib did not confer the anticipated clinical benefits for advanced SCLC patients harboring genetic alterations within this pathway [73].

### PI3Kα inhibitors

In order to improve the therapeutic efficacy in clinical trial, more selective PI3k inhibitors were developed based on the advances in medicinal chemistry and a deeper understanding of PI3K isoform-specific functions. PI3Kα-specific inhibitors, such as BYL719/alpelisib and GDC-0032/taselisib, have demonstrated efficacy in preclinical models by suppressing downstream signaling pathways and inhibiting tumor growth [56, 74]. Alpelisib, which is approved for treatment of breast cancer with PIK3CA mutations, has shown antitumor activity in patient-derived cells via suppressing the expression of retinoblastoma-associated protein and tumor suppressor protein p53 [75]. Meanwhile, a phase II clinical study has demonstrated that alpelisib exhibited a commendable antitumor efficacy in NSCLC patients with a favorable safety profile [76]. Similarly, taselisib exhibited significant tumor regression in PIK3CA-mutant lung cancer [77]. In squamous cell lung cancer, though taselisib has failed to meet primary endpoint in phase II trial [78, 79], it still showed promising anti-lung cancer potential by MEK inhibitor in vitro [80].

Serabelisib is another PI3Kα-specific inhibitor. A phase I clinical trial of serabelisib in a cohort comprising patients with advanced NSCLC has revealed that an intermittent dosing regimen of serabelisib was associated with an acceptable safety profile. The findings suggested that intermittent dosing may enhance the therapeutic index of serabelisib by potentially mitigating dose-limiting toxicities while maintaining antitumor activity. Despite the study indicating a limited efficacy for serabelisib as a monotherapy, the encouraging safety and pharmacokinetic profile of serabelisib warrant further exploration in combination therapies [81].

### PI3Kβ inhibitors

PI3Kβ inhibitors, such as GSK2636771 and AZD8186, have shown potential in inhibiting tumor growth in preclinical lung cancer. It was found that orally bioavailable GSK2636771 showed a favorable safety profile in clinical trial and lung cancer patients who received GSK2636771 remained free of progression for 33 weeks [82]. Further study revealed that AZD8186 was a dual inhibitor of PI3Kβ and PI3Kδ [83]. It was reported to show acceptable safety and tolerability profile in treating PTEN deficient solid tumors, including squamous cell lung cancer [84, 85].

### PI3Kγ inhibitor

Eganelisib is a representative PI3Kγ-specific inhibitor. To date, there have been no clinical trials specifically targeting lung cancer with eganelisib. However, the potential therapeutic effects of the compound on lung malignancies are under investigation. It was found that eganelisib significantly suppressed lung cancer progression in animal model [86]. Other novel PI3Kγ-specific inhibitor, such as MTX-531, was shown to inhibit cancer progression while not lead to the hyperglycemia via weak activating peroxisome proliferator-activated receptor-γ. However, the function of MTX-531 has not been studied in lung cancer [56].

### PI3Kδ inhibitors

PI3Kδ facilitates immune evasion by suppressing cytotoxic T-cell responses and promoting regulatory T-cell (Treg) expansion, which dampens anti-tumor immunity [36]. Inhibitors targeting PI3Kδ, such as CAL-101/Idelalisib, have been explored for their potential to enhance immune-mediated tumor clearance via direct inhibition on the function of Tregs and myeloid derived suppressor cells (MDSC) [87]. However, a phase Ib/II trial assessing the safety and efficacy of idelalisib in NSCLC patients after PD-1 blockade was discontinued due to inadequate participant enrollment, compromising the study’s statistical validity (NCT03257722). Umbralisib is a PI3Kδ-specific inhibitor that has been extensively studied in the context of leukemia. Studies have demonstrated that umbralisib is well tolerated and exhibits efficacy against relapsed or refractory hematological cancers [88]. While the antitumor capacity of umbralisib in lung cancer has not been studied yet. TQ-B3525 is another dual PI3Kα/δ inhibitor which has been progressed to phase II clinical trials that focused on NSCLC patients (NCT05284994).

### Mechanisms underlying PI3K inhibitors in lung cancer

PI3K inhibitors exert antitumor effects through multiple, interconnected mechanisms within the PI3K/AKT/mTOR pathway:

- Direct enzyme inhibition: Selective inhibitors bind to the PI3K catalytic subunits, preventing the conversion of PIP2 to PIP3, thereby blocking AKT activation [89].
- Suppression of AKT activation: By inhibiting PIP3 production, these inhibitors reduce AKT phosphorylation, thereby diminishing cell growth and survival signaling [90].
- Downregulation of mTOR Signaling: Inhibition of AKT leads to decreased mTOR activity [91], impairing protein synthesis, ribosome biogenesis, and cell cycle progression, which reduces cellular proliferation.
- Induction of apoptosis: PI3K inhibitors can trigger programmed cell death by inhibiting anti-apoptotic proteins such as BCL-xL [92], leading to caspase activation.
- Inhibition of angiogenesis: These inhibitors downregulate VEGF expression [93], thereby limiting the formation of new blood vessels essential for tumor growth.
- Cell cycle modulation: PI3K inhibitors interfere with cyclin-dependent kinases (CDKs), thereby causing cell cycle arrest and reduced proliferation [94].

These multifaceted mechanisms highlight the potential of PI3K inhibitors as effective therapeutic agents in lung cancer. However, the complexity and redundancy of cellular signaling networks can activate compensatory pathways, potentially undermining the long-term efficacy of these inhibitors.

### Mechanisms underlying resistance to PI3K inhibitors

Despite their therapeutic promise, the clinical efficacy of PI3K inhibitors is often compromised by the development of resistance. Understanding these resistance mechanisms is crucial for developing effective counterstrategies.

- Activation of alternative pathways: Lung cancer cells may activate compensatory signaling pathways, such as the MAPK/ERK axis, to sustain proliferation and survival despite PI3K inhibition [95]. Meanwhile, the janus kinase/signal transducer and activator of transcription (JAK/STAT) pathway can also be upregulated in response to PI3K inhibition. Activation of JAK/STAT signaling contributes to resistance by promoting gene transcription that supports cell growth and survival [96]. These exemplary alternative pathways play a pivotal role in bolstering cellular survival and proliferation, effectively equipping cancer cells with the wherewithal to circumvent the therapeutic blockades imposed by PI3K inhibitors.
- Genetic alterations: Activating mutations in the catalytic subunit of PI3K, such as PIK3CA and PIK3CB, result in hyperactivation of the PI3K/Akt/mTOR signaling pathway. Such mutations enhance the survival and proliferation of cancer cells, thereby conferring resistance to PI3K inhibitors [19, 97]. Additionally, mutations in downstream effectors like AKT can sustain pathway activation despite PI3K inhibition [98]. PTEN is a critical negative regulator of the PI3K pathway. Loss or functional inactivation of PTEN leads to unchecked PI3K signaling, thereby diminishing the efficacy of PI3K inhibitors [99]. Tumors with PTEN deficiency often exhibit persistent PI3K pathway activity despite therapeutic intervention [24].
- Metabolic reprogramming: Cancer cells often undergo metabolic reprogramming to adapt to therapeutic pressures. Enhanced glycolysis (the Warburg effect) and alterations in other metabolic pathways can provide the necessary energy and biosynthetic precursors to sustain cell growth despite PI3K pathway inhibition [100]. Metabolic flexibility allows cancer cells to circumvent the inhibitory effects of PI3K-targeted treatments [101].
- Feedback activation mechanism: PI3K inhibitors can disrupt negative feedback loops that normally restrain upstream or parallel signaling molecules. For instance, inhibition of the PI3K pathway may relieve suppression of receptor tyrosine kinases such as HER3 and insulin-like growth factor 1 receptor (IGF-1R). Elevated expression and activation of these receptors can reactivate downstream signaling pathways, thus attenuating the therapeutic effects of PI3K inhibitors [102, 103].

Addressing resistance necessitates a multifaceted approach. Combination therapies targeting multiple pathways are essential to prevent compensatory mechanisms. Additionally, personalized medicine strategies involving molecular profiling of tumors can identify specific resistance mechanisms, allowing for tailored therapeutic interventions. Future directions emphasize the integration of personalized medicine and combination therapies to overcome resistance and improve outcomes for lung cancer patients. As our understanding of PI3K biology deepens, PI3K inhibitors hold promise as integral components of comprehensive lung cancer treatment regimens.

## Discussion

Despite significant advancements in elucidating the role of PI3K signaling in lung cancer, several critical research gaps and limitations persist, impeding the translation of these insights into effective therapies. A comprehensive understanding of PI3K subtypes remains incomplete, particularly concerning PI3Kγ and PI3Kδ. While PI3Kα and PI3Kβ have been extensively characterized, the specific functions and interactions of PI3Kγ and PI3Kδ with other signaling pathways in lung cancer are not fully elucidated. This knowledge deficit hinders the development of targeted therapies that could potentially exploit these subtypes’ unique roles within oncogenic processes.

Another major challenge is the limited efficacy of current PI3K inhibitors in clinical settings. Although these inhibitors have shown promise in preclinical models, their clinical outcomes have been modest. The primary reasons include the emergence of resistance mechanisms and the inherent heterogeneity of lung tumors [29, 104]. Additionally, the absence of robust biomarkers to predict patient response and the variability in PI3K pathway activation across different lung cancer subtypes contribute to inconsistent therapeutic efficacy. This underscores the necessity for more personalized approaches in targeting the PI3K pathway.

The TME further complicates the efficacy of PI3K inhibitors. Interactions within the TME, including immune cell infiltration, stromal interactions, and metabolic support, significantly influence therapeutic outcomes. Current preclinical models do not adequately capture these complex interactions, limiting the translatability of findings to clinical success. Moreover, the adverse effects associated with PI3K inhibitors, such as hyperglycemia, hypertension, and immunosuppression, pose significant challenges. These side effects can adversely affect patient quality of life and adherence to treatment regimens, necessitating the development of strategies to manage and mitigate these adverse outcomes.

Addressing these challenges requires a multifaceted approach focusing on several key areas. Firstly, further investigation into the distinct roles of each PI3K isoform in various lung cancer subtypes and stages is imperative. Detailed studies elucidating subtype-specific functions will facilitate the development of more precise and effective targeted therapies [105]. Additionally, gaining mechanistic insights into how PI3K subtypes interact with other signaling pathways and contribute to processes such as metastasis, immune modulation, and metabolic reprogramming will enable the identification of novel therapeutic targets and the design of combination strategies.

Biomarker development is equally crucial. Identifying subtype-specific biomarkers for prognosis and treatment response will allow for personalized therapeutic approaches, ensuring that patients receive treatments tailored to their specific tumor profiles. In parallel, the development of novel PI3K inhibitors with higher specificity and the ability to overcome resistance mechanisms is essential. Rational drug design, including structure-based approaches and high-throughput screening, can lead to the creation of inhibitors that minimize off-target effects while enhancing therapeutic efficacy. Furthermore, next-generation inhibitors that can bypass or counteract known resistance mechanisms, such as mutations in PI3K isoforms or activation of compensatory pathways, hold promise for improving clinical outcomes.

Combination therapy strategies also present a viable path forward. Combining PI3K inhibitors with other targeted agents, such as EGFR tyrosine kinase inhibitors or ALK inhibitors, can help overcome resistance in specific lung cancer subtypes. Additionally, integrating PI3K inhibition with immunotherapies, such as immune checkpoint inhibitors, may enhance antitumor immunity by modulating the immune response while simultaneously targeting tumor signaling pathways. These synergistic combinations could potentiate antitumor activity and prevent the emergence of resistance, thereby improving overall therapeutic efficacy.

Personalized medicine approaches, underpinned by comprehensive genetic and molecular profiling, are critical for optimizing PI3K-targeted therapies. Utilizing genetic profiling to tailor treatments based on specific mutations, such as PIK3CA mutations, ensures that patients receive the most effective therapies for their unique tumor characteristics. Moreover, selecting PI3K inhibitors based on the predominant PI3K subtype alterations in individual tumors enables more precise targeting of oncogenic pathways, reducing off-target effects and enhancing therapeutic outcomes. Comprehensive treatment regimens that combine PI3K inhibitors with other therapies, informed by metabolic and immune profiling of tumors, can address the diverse aspects of tumor biology, thereby enhancing therapeutic efficacy and minimizing resistance.

## Conclusion

While significant strides have been made in understanding PI3K signaling in lung cancer, addressing the existing research gaps through targeted investigations, novel inhibitor development, combination therapies, and personalized medicine approaches will be pivotal in advancing PI3K-targeted therapies from modest clinical efficacy to meaningful patient outcomes.

**Fig. 1:**
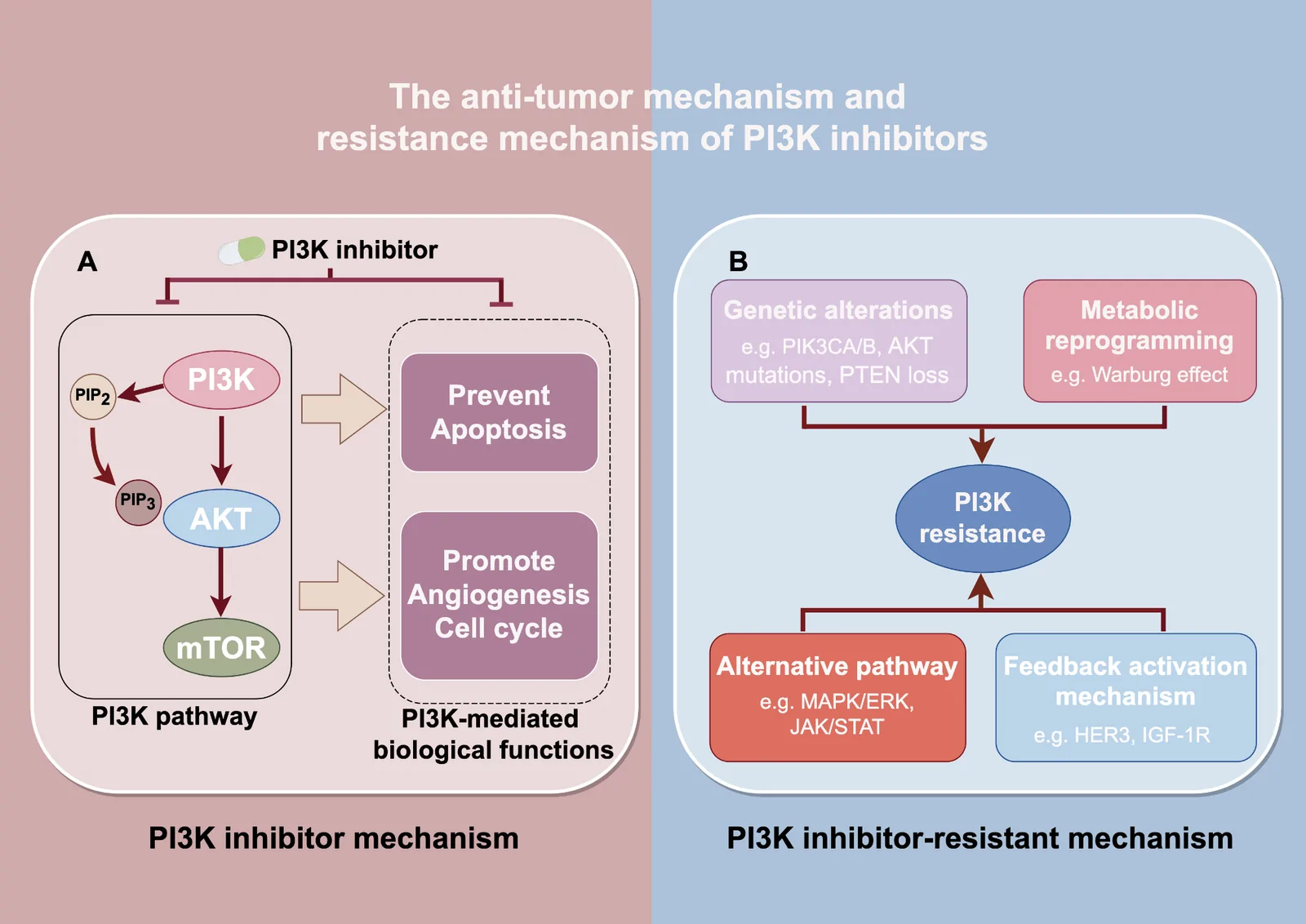
Therapeutic and resistance mechanisms of PI3K inhibitors in lung cancer treatment. (**A**) PI3K inhibitor directly inhibit PI3K/AKT/mTOR pathway and consequently promote apoptosis and prevent angiogenesis and aberrant cell cycle. (**B**) Major reasons contribute to drug resistant of PI3K inhibitor

**Fig. 2:**
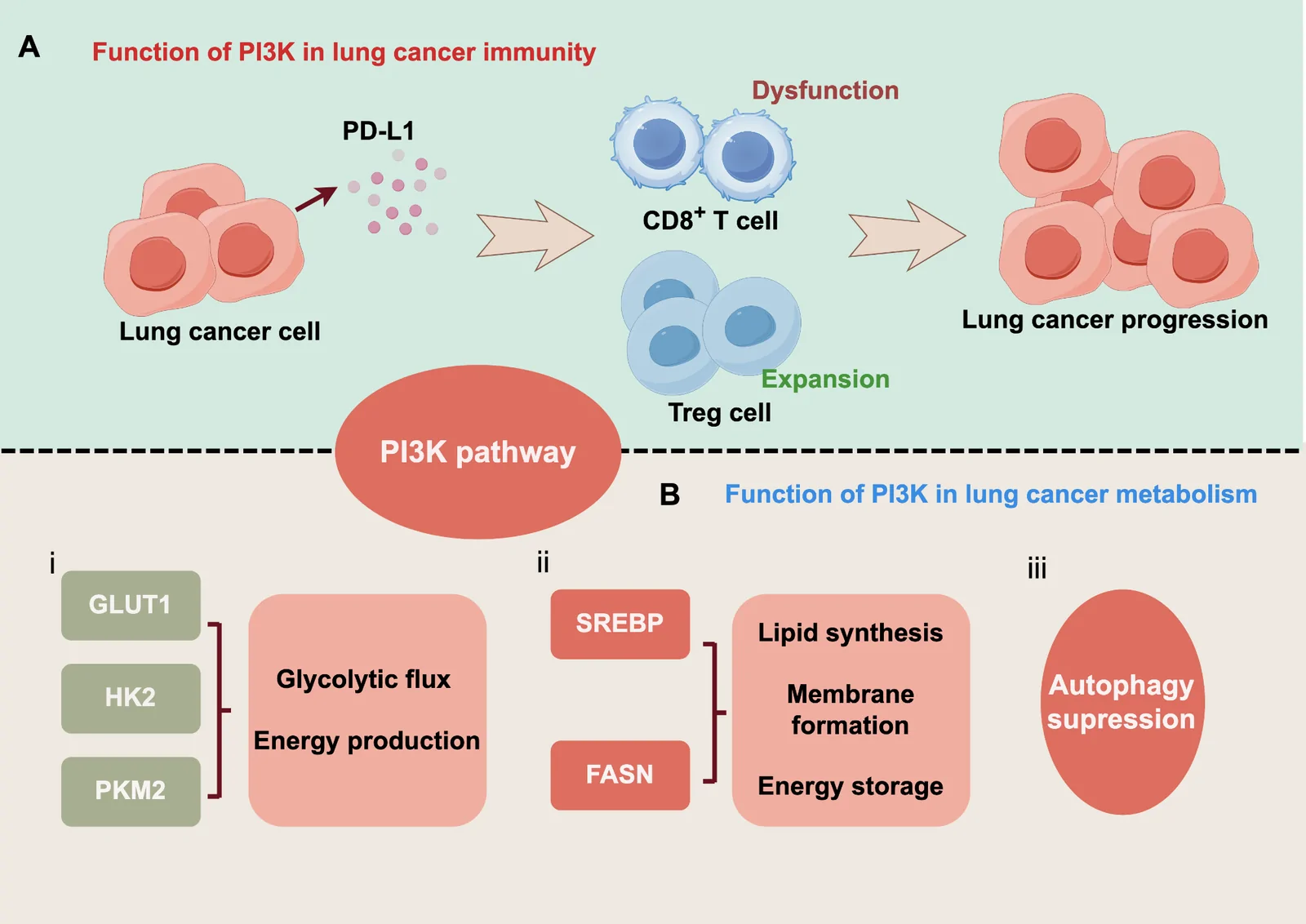
Function of PI3K in immunity and metabolism regulation in lung cancer. (**A**) Function of PI3K in lung cancer immunity. (**B**) Function of PI3K in lung cancer metabolism

## Electronic supplementary material

Below is the link to the electronic supplementary material.

Supplementary Material 1

Supplementary Material 2

## Data Availability

The data presented in this study are available on request from the corresponding author.

## Abbreviations

EGFR: Epidermal growth factor receptor
EMT: Epithelial-to-mesenchymal transition
FDA: Food and Drug Administration
GLUT1: Glucose transporter 1
HER2: Human epidermal growth factor receptor 2
HK2: Hexokinase 2
ICIs: Immune checkpoint inhibitors
IGF-1R: Insulin-like growth factor 1 receptor
JAK/STAT: Janus kinase/signal transducer and activator of transcription
MDSC: Myeloid derived suppressor cells
mTOR: Mechanistic target of rapamycin
PD-L1: Programmed death-ligand 1
PH: Pleckstrin homology
PI3K: Phosphatidylinositol 3-kinase
PIP2: Phosphatidylinositol-4,5-bisphosphate
PIP3: Phosphatidylinositol-3,4,5-trisphosphate
PKM2: Pyruvate kinase M2
PTEN: Phosphatase and tensin homolog
RTKs: Receptor tyrosine kinases
SREBPs: Sterol regulatory element-binding proteins
TAMs: Tumor-associated macrophages
TME: Tumor microenvironment
Treg: Regulatory T-cell

## References

[1] Siegel RL, Giaquinto AN, Jemal A. Cancer statistics, 2024. Cancer J Clin. 2024;74(1):12–49. 10.3322/caac.21820 38230766

[2] Li Y, Yan B, He S. Advances and challenges in the treatment of lung cancer. Biomed Pharmacother. 2023;169:115891. 37979378 10.1016/j.biopha.2023.115891

[3] Samarth N, Gulhane P, Singh S. Immunoregulatory framework and the role of miRNA in the pathogenesis of NSCLC - A systematic review. Front Oncol. 2022;12:1089320. 36620544 10.3389/fonc.2022.1089320 PMC9811680

[4] Ji M, Wang D, Lin S, Wang C, Li L, Zhang Z, et al. A novel PI3K inhibitor XH30 suppresses orthotopic glioblastoma and brain metastasis in mice models. Acta Pharm Sin B. 2022;12(2):774–86. 35256946 10.1016/j.apsb.2021.05.019 PMC8897175

[5] Mallick S, Duttaroy AK, Dutta S. The PIK3CA gene and its pivotal role in tumor tropism of triple-negative breast cancer. Translational Oncol. 2024;50:102140. 10.1016/j.tranon.2024.102140 PMC11491976 39369580

[6] Bergholz JS, Wang Q, Wang Q, Ramseier M, Prakadan S, Wang W, et al. PI3Kβ controls immune evasion in PTEN-deficient breast tumours. Nature. 2023;617(7959):139–46. 37076617 10.1038/s41586-023-05940-w PMC10494520

[7] Xu H, Russell SN, Steiner K, O’Neill E, Jones KI. Targeting PI3K-gamma in myeloid driven tumour immune suppression: a systematic review and meta-analysis of the preclinical literature. Cancer Immunol Immunother. 2024;73(10):204. 39105848 10.1007/s00262-024-03779-2 PMC11303654

[8] Muniz-Bongers LR, McClain CB, Saxena M, Bongers G, Merad M, Bhardwaj N. MMP2 and TLRs modulate immune responses in the tumor microenvironment. JCI Insight. 2021;6(12). 10.1172/jci.insight.144913 PMC8262464 34032639

[9] Liu P, Cheng H, Roberts TM, Zhao JJ. Targeting the phosphoinositide 3-kinase pathway in cancer. Nat Rev Drug Discov. 2009;8(8):627–44. 19644473 10.1038/nrd2926 PMC3142564

[10] Lien EC, Lyssiotis CA, Cantley LC. Metabolic reprogramming by the PI3K-Akt-mTOR pathway in Cancer. Recent Results Cancer Res. 2016;207:39–72. 27557534 10.1007/978-3-319-42118-6_3

[11] Yu M, Chen J, Xu Z, Yang B, He Q, Luo P, et al. Development and safety of PI3K inhibitors in cancer. Arch Toxicol. 2023;97(3):635–50. 36773078 10.1007/s00204-023-03440-4 PMC9968701

[12] Jean S, Kiger AA. Classes of phosphoinositide 3-kinases at a glance. J Cell Sci. 2014;127(Pt 5):923–8. 24587488 10.1242/jcs.093773 PMC3937771

[13] He Y, Sun MM, Zhang GG, Yang J, Chen KS, Xu WW, et al. Targeting PI3K/Akt signal transduction for cancer therapy. Signal Transduct Target Ther. 2021;6(1):425. 34916492 10.1038/s41392-021-00828-5 PMC8677728

[14] Xu Y, Afify SM, Du J, Liu B, Hassan G, Wang Q, et al. The efficacy of PI3Kγ and EGFR inhibitors on the suppression of the characteristics of cancer stem cells. Sci Rep. 2022;12(1):347. 35013447 10.1038/s41598-021-04265-w PMC8748513

[15] Yamamoto H, Shigematsu H, Nomura M, Lockwood WW, Sato M, Okumura N, et al. PIK3CA mutations and copy number gains in human lung cancers. Cancer Res. 2008;68(17):6913–21. 18757405 10.1158/0008-5472.CAN-07-5084 PMC2874836

[16] Wang Y, Wang Y, Li J, Li J, Che G. Clinical significance of PIK3CA Gene in Non-small-cell Lung Cancer: a systematic review and Meta-analysis. Biomed Res Int. 2020;2020:3608241. 32908885 10.1155/2020/3608241 PMC7450343

[17] Li J, Kaneda MM, Ma J, Li M, Shepard RM, Patel K et al. PI3Kγ inhibition suppresses microglia/TAM accumulation in glioblastoma microenvironment to promote exceptional temozolomide response. Proceedings of the National Academy of Sciences. 2021;118(16):e2009290118. 10.1073/pnas.2009290118 PMC8072253 33846242

[18] Peng Y, Wang Y, Zhou C, Mei W, Zeng C. PI3K/Akt/mTOR pathway and its role in Cancer therapeutics: are we making Headway? Front Oncol. 2022;12:819128. 35402264 10.3389/fonc.2022.819128 PMC8987494

[19] Liu X, Mei W, Zhang P, Zeng C. PIK3CA mutation as an acquired resistance driver to EGFR-TKIs in non-small cell lung cancer: clinical challenges and opportunities. Pharmacol Res. 2024;202:107123. 38432445 10.1016/j.phrs.2024.107123

[20] Cuesta C, Arévalo-Alameda C, Castellano E. The importance of being PI3K in the RAS Signaling Network. Genes (Basel). 2021;12(7). 10.3390/genes12071094 PMC8303222 34356110

[21] Eng J, Woo KM, Sima CS, Plodkowski A, Hellmann MD, Chaft JE, et al. Impact of concurrent PIK3CA mutations on response to EGFR Tyrosine Kinase Inhibition in EGFR-Mutant Lung cancers and on prognosis in Oncogene-Driven Lung Adenocarcinomas. J Thorac Oncol. 2015;10(12):1713–9. 26334752 10.1097/JTO.0000000000000671 PMC4760768

[22] Chakrabarty A, Rexer BN, Wang SE, Cook RS, Engelman JA, Arteaga CL. H1047R phosphatidylinositol 3-kinase mutant enhances HER2-mediated transformation by heregulin production and activation of HER3. Oncogene. 2010;29(37):5193–203. 20581867 10.1038/onc.2010.257 PMC2945381

[23] Sirhan Z, Alojair R, Thyagarajan A, Sahu RP. Therapeutic implications of PTEN in Non-small Cell Lung Cancer. Pharmaceutics. 2023;15(8). 10.3390/pharmaceutics15082090 PMC10458395 37631304

[24] Vidotto T, Melo CM, Castelli E, Koti M, dos Reis RB, Squire JA. Emerging role of PTEN loss in evasion of the immune response to tumours. Br J Cancer. 2020;122(12):1732–43. 32327707 10.1038/s41416-020-0834-6 PMC7283470

[25] Hu Z-Y, Huang W-Y, Zhang L, Huang B, Chen S-C, Li X-L. Expression of AKT and p-AKT protein in lung adenocarcinoma and its correlation with PD-L1 protein and prognosis. Annals Translational Med. 2020;8(18):1172. 10.21037/atm-20-5865 PMC7576079 33241021

[26] Chouhan S, Kumar A, Piprode V, Dasgupta A, Singh S, Khalique A. Regulatory-Associated protein of mTOR-Mediated signaling: a Nexus between Tumorigenesis and Disease. Targets. 2024;2(4):341–71.

[27] Glaviano A, Foo ASC, Lam HY, Yap KCH, Jacot W, Jones RH, et al. PI3K/AKT/mTOR signaling transduction pathway and targeted therapies in cancer. Mol Cancer. 2023;22(1):138. 37596643 10.1186/s12943-023-01827-6 PMC10436543

[28] Liu Q, Yu S, Zhao W, Qin S, Chu Q, Wu K. EGFR-TKIs resistance via EGFR-independent signaling pathways. Mol Cancer. 2018;17(1):53. 29455669 10.1186/s12943-018-0793-1 PMC5817859

[29] Yang J, Nie J, Ma X, Wei Y, Peng Y, Wei X. Targeting PI3K in cancer: mechanisms and advances in clinical trials. Mol Cancer. 2019;18(1):26. 30782187 10.1186/s12943-019-0954-x PMC6379961

[30] Dong C, Wu J, Chen Y, Nie J, Chen C. Activation of PI3K/AKT/mTOR pathway causes drug resistance in breast Cancer. Front Pharmacol. 2021;12. 10.3389/fphar.2021.628690 PMC8005514 33790792

[31] Li Q, Geng S, Luo H, Wang W, Mo Y-Q, Luo Q, et al. Signaling pathways involved in colorectal cancer: pathogenesis and targeted therapy. Signal Transduct Target Therapy. 2024;9(1):266. 10.1038/s41392-024-01953-7 PMC11456611 39370455

[32] Jiang X, Wang J, Deng X, Xiong F, Ge J, Xiang B, et al. Role of the tumor microenvironment in PD-L1/PD-1-mediated tumor immune escape. Mol Cancer. 2019;18(1):10. 30646912 10.1186/s12943-018-0928-4 PMC6332843

[33] Cui J-W, Li Y, Yang Y, Yang H-K, Dong J-M, Xiao Z-H, et al. Tumor immunotherapy resistance: revealing the mechanism of PD-1 / PD-L1-mediated tumor immune escape. Biomed Pharmacother. 2024;171:116203. 38280330 10.1016/j.biopha.2024.116203

[34] Yazdimamaghani M, Kolupaev OV, Lim C, Hwang D, Laurie SJ, Perou CM, et al. Tumor microenvironment immunomodulation by nanoformulated TLR 7/8 agonist and PI3k delta inhibitor enhances therapeutic benefits of radiotherapy. Biomaterials. 2025;312:122750. 39126779 10.1016/j.biomaterials.2024.122750 PMC11401478

[35] Sun P, Meng LH. Emerging roles of class I PI3K inhibitors in modulating tumor microenvironment and immunity. Acta Pharmacol Sin. 2020;41(11):1395–402. 32939035 10.1038/s41401-020-00500-8 PMC7656798

[36] Okkenhaug K, Graupera M, Vanhaesebroeck B. Targeting PI3K in Cancer: impact on Tumor cells, their protective stroma, angiogenesis, and Immunotherapy. Cancer Discov. 2016;6(10):1090–105. 27655435 10.1158/2159-8290.CD-16-0716 PMC5293166

[37] Funk CR, Wang S, Chen KZ, Waller A, Sharma A, Edgar CL, et al. PI3Kδ/γ inhibition promotes human CART cell epigenetic and metabolic reprogramming to enhance antitumor cytotoxicity. Blood. 2022;139(4):523–37. 35084470 10.1182/blood.2021011597 PMC8796652

[38] Castel P, Toska E, Engelman JA, Scaltriti M. The present and future of PI3K inhibitors for cancer therapy. Nat Cancer. 2021;2(6):587–97. 35118422 10.1038/s43018-021-00218-4 PMC8809509

[39] Yin Q, Wu L, Han L, Zheng X, Tong R, Li L, et al. Immune-related adverse events of immune checkpoint inhibitors: a review. Front Immunol. 2023;14:1167975. 37304306 10.3389/fimmu.2023.1167975 PMC10247998

[40] Faehling S, Coelho M, Floerchinger A, Schneider C, Stilgenbauer S, Lichter P, et al. Simultaneous inhibition of PI3Kgamma and PI3Kdelta deteriorates T-cell function with implications for chronic lymphocytic leukemia. Hemasphere. 2023;7(3):e840. 36844182 10.1097/HS9.0000000000000840 PMC9949793

[41] Heavey S, Dowling P, Moore G, Barr MP, Kelly N, Maher SG, et al. Development and characterisation of a panel of phosphatidylinositide 3-kinase– mammalian target of rapamycin inhibitor resistant lung cancer cell lines. Sci Rep. 2018;8(1):1652. 29374181 10.1038/s41598-018-19688-1 PMC5786033

[42] Deng H, Chen Y, Li P, Hang Q, Zhang P, Jin Y, et al. PI3K/AKT/mTOR pathway, hypoxia, and glucose metabolism: potential targets to overcome radioresistance in small cell lung cancer. Cancer Pathog Ther. 2023;1(1):56–66. 38328610 10.1016/j.cpt.2022.09.001 PMC10846321

[43] Han B, Lin X, Hu H. Regulation of PI3K signaling in cancer metabolism and PI3K-targeting therapy. Transl Breast Cancer Res. 2024;5:33. 39534586 10.21037/tbcr-24-29 PMC11557167

[44] Tantai J, Pan X, Chen Y, Shen Y, Ji C. TRIM46 activates AKT/HK2 signaling by modifying PHLPP2 ubiquitylation to promote glycolysis and chemoresistance of lung cancer cells. Cell Death Dis. 2022;13(3):285. 35354796 10.1038/s41419-022-04727-7 PMC8967906

[45] Krycer JR, Sharpe LJ, Luu W, Brown AJ. The Akt-SREBP nexus: cell signaling meets lipid metabolism. Trends Endocrinol Metab. 2010;21(5):268–76. 20117946 10.1016/j.tem.2010.01.001

[46] Tufail M, Jiang C-H, Li N. Altered metabolism in cancer: insights into energy pathways and therapeutic targets. Mol Cancer. 2024;23(1):203. 39294640 10.1186/s12943-024-02119-3 PMC11409553

[47] Majem B, Nadal E, Muñoz-Pinedo C. Exploiting metabolic vulnerabilities of non small cell lung carcinoma. Semin Cell Dev Biol. 2020;98:54–62. 31238096 10.1016/j.semcdb.2019.06.004

[48] Zhuo B, Li Y, Li Z, Qin H, Sun Q, Zhang F, et al. PI3K/Akt signaling mediated Hexokinase-2 expression inhibits cell apoptosis and promotes tumor growth in pediatric osteosarcoma. Biochem Biophys Res Commun. 2015;464(2):401–6. 26116768 10.1016/j.bbrc.2015.06.092

[49] Zahra K, Dey T, Ashish, Mishra SP, Pandey U. Pyruvate kinase M2 and Cancer: the role of PKM2 in promoting Tumorigenesis. Front Oncol. 2020;10:159. 32195169 10.3389/fonc.2020.00159 PMC7061896

[50] Xiao Y, Yang Y, Xiong H, Dong G. The implications of FASN in immune cell biology and related diseases. Cell Death Dis. 2024;15(1):88. 38272906 10.1038/s41419-024-06463-6 PMC10810964

[51] Marijt KA, Sluijter M, Blijleven L, Tolmeijer SH, Scheeren FA, van der Burg SH, et al. Metabolic stress in cancer cells induces immune escape through a PI3K-dependent blockade of IFNγ receptor signaling. J Immunother Cancer. 2019;7(1):152. 31196219 10.1186/s40425-019-0627-8 PMC6567539

[52] Tang F, Zhang J-N, Zhao X-L, Xu L-Y, Ao H, Peng C. Unlocking the dual role of autophagy: a new strategy for treating lung cancer. J Pharm Anal. 2024:101098. 10.1016/j.jpha.2024.101098 PMC11919427 40104173

[53] Shanshan W, Hongying M, Jingjing F, Rui Y. Metformin and buparlisib synergistically induce apoptosis of non-small lung cancer (NSCLC) cells via Akt/FoxO3a/Puma axis. Toxicol Vitro. 2024;97:105801. 10.1016/j.tiv.2024.105801 38479708

[54] Wu Y-Y, Wu H-C, Wu J-E, Huang K-Y, Yang S-C, Chen S-X, et al. The dual PI3K/mTOR inhibitor BEZ235 restricts the growth of lung cancer tumors regardless of EGFR status, as a potent accompanist in combined therapeutic regimens. J Experimental Clin Cancer Res. 2019;38(1):282. 10.1186/s13046-019-1282-0 PMC6604380 31262325

[55] Zhang BT, Leung PC, Wong CK, Wang DJ. The Immunomodulatory effects of vitamin D on COVID-19 Induced Glioblastoma Recurrence via the PI3K-AKT signaling pathway. Int J Mol Sci. 2024;25(23). 10.3390/ijms252312952 PMC11641820 39684661

[56] Whitehead CE, Ziemke EK, Frankowski-McGregor CL, Mumby RA, Chung J, Li J, et al. A first-in-class selective inhibitor of EGFR and PI3K offers a single-molecule approach to targeting adaptive resistance. Nat Cancer. 2024;5(8):1250–66. 38992135 10.1038/s43018-024-00781-6 PMC11357990

[57] Zeng M, Hu Y, Zhao L, Duan C, Wu H, Xu Y, et al. Design, synthesis, and pharmacological evaluation of triazine-based PI3K/mTOR inhibitors for the potential treatment of non-small cell lung cancer. Eur J Med Chem. 2024;284:117200. 39733482 10.1016/j.ejmech.2024.117200

[58] Lin F, Shen J, Li H, Liu L. β-carboline compound-10830733 suppresses the progression of non-small cell lung cancer by inhibiting the PI3K/Akt/GSK 3β signaling pathway. Eur J Pharmacol. 2025;986:177131. 39566811 10.1016/j.ejphar.2024.177131

[59] Tanaka H, Yoshida M, Tanimura H, Fujii T, Sakata K, Tachibana Y, et al. The selective class I PI3K inhibitor CH5132799 targets human cancers harboring oncogenic PIK3CA mutations. Clin Cancer Res. 2011;17(10):3272–81. 21558396 10.1158/1078-0432.CCR-10-2882

[60] Zhou H, Yu C, Kong L, Xu X, Yan J, Li Y, et al. B591, a novel specific pan-PI3K inhibitor, preferentially targets cancer stem cells. Oncogene. 2019;38(18):3371–86. 30635656 10.1038/s41388-018-0674-5 PMC6756013

[61] Roskoski R. Properties of FDA-approved small molecule protein kinase inhibitors: a 2024 update. Pharmacol Res. 2024;200:107059. 38216005 10.1016/j.phrs.2024.107059

[62] Abdallah ME, El-Readi MZ, Althubiti MA, Almaimani RA, Ismail AM, Idris S, et al. Tamoxifen and the PI3K inhibitor: LY294002 synergistically induce apoptosis and cell cycle arrest in breast Cancer MCF-7 cells. Molecules. 2020;25(15):3355. 32722075 10.3390/molecules25153355 PMC7436112

[63] Kong D, Yamori T. Phosphatidylinositol 3-kinase inhibitors: promising drug candidates for cancer therapy. Cancer Sci. 2008;99(9):1734–40. 18616528 10.1111/j.1349-7006.2008.00891.x PMC11160043

[64] Ihle NT, Paine-Murrieta G, Berggren MI, Baker A, Tate WR, Wipf P, et al. The phosphatidylinositol-3-kinase inhibitor PX-866 overcomes resistance to the epidermal growth factor receptor inhibitor gefitinib in A-549 human non-small cell lung cancer xenografts. Mol Cancer Ther. 2005;4(9):1349–57. 16170026 10.1158/1535-7163.MCT-05-0149 PMC1432090

[65] Koul D, Shen R, Kim YW, Kondo Y, Lu Y, Bankson J, et al. Cellular and in vivo activity of a novel PI3K inhibitor, PX-866, against human glioblastoma. Neuro Oncol. 2010;12(6):559–69. 20156803 10.1093/neuonc/nop058 PMC2940638

[66] Levy B, Spira A, Becker D, Evans T, Schnadig I, Camidge DR, et al. A randomized, phase 2 trial of Docetaxel with or without PX-866, an irreversible oral phosphatidylinositol 3-kinase inhibitor, in patients with relapsed or metastatic non-small-cell lung cancer. J Thorac Oncol. 2014;9(7):1031–5. 24926548 10.1097/JTO.0000000000000183

[67] Soria J-C, LoRusso P, Bahleda R, Lager J, Liu L, Jiang J, et al. Phase I dose-escalation study of Pilaralisib (SAR245408, XL147), a Pan-class I PI3K inhibitor, in Combination with Erlotinib in patients with solid tumors. Oncologist. 2015;20(3):245–6. 25669662 10.1634/theoncologist.2014-0449 PMC4350809

[68] Edelman G, Rodon J, Lager J, Castell C, Jiang J, Van Allen EM, et al. Phase I trial of a Tablet Formulation of Pilaralisib, a Pan-class I PI3K inhibitor, in patients with Advanced Solid tumors. Oncologist. 2018;23(4):401–e38. 29593099 10.1634/theoncologist.2017-0691 PMC5896717

[69] Besse B, Luft AV, Fadeeva N, Mezger J, Beck T, Bidoli P, et al. editors. A phase II trial of pictilisib with chemotherapy in first-line non-squamous NSCLC. ELSEVIER SCIENCE INC 360 PARK AVE SOUTH. NEW YORK, NY 10010– 1710 USA: JOURNAL OF THORACIC ONCOLOGY; 2015.

[70] Mallon R, Feldberg LR, Lucas J, Chaudhary I, Dehnhardt C, Santos ED, Chen Z, dos Santos O, Ayral-Kaloustian S, Venkatesan A, Hollander I. Antitumor efficacy of PKI-587, a highly potent dual PI3K/mTOR kinase inhibitor. Clin Cancer Res. 2011;17(10):3193–203. 21325073 10.1158/1078-0432.CCR-10-1694

[71] Venkatesan AM, Dehnhardt CM, Delos Santos E, Chen Z, Dos Santos O, Ayral-Kaloustian S, et al. Bis(morpholino-1,3,5-triazine) derivatives: potent adenosine 5’-triphosphate competitive phosphatidylinositol-3-kinase/mammalian target of rapamycin inhibitors: discovery of compound 26 (PKI-587), a highly efficacious dual inhibitor. J Med Chem. 2010;53(6):2636–45. 20166697 10.1021/jm901830p

[72] Makinoshima H, Umemura S, Suzuki A, Nakanishi H, Maruyama A, Udagawa H, et al. Metabolic determinants of sensitivity to Phosphatidylinositol 3-Kinase pathway inhibitor in small-cell lung carcinoma. Cancer Res. 2018;78(9):2179–90. 29490947 10.1158/0008-5472.CAN-17-2109

[73] Udagawa H, Ikeda T, Umemura S, Daga H, Toyozawa R, Harada D, et al. Phase II study of gedatolisib for small-cell lung cancer (SCLC) patients (pts) with genetic alterations in PI3K/AKT/mTOR pathway based on a large-scale nationwide genomic screening network in Japan (EAGLE-PAT/LC-SCRUM-Japan). J Clin Oncol. 2020;38(15suppl):9064.

[74] Verret B, Cortes J, Bachelot T, Andre F, Arnedos M. Efficacy of PI3K inhibitors in advanced breast cancer. Ann Oncol. 2019;30:x12–20. 31859349 10.1093/annonc/mdz381 PMC6923787

[75] Yu N, Hwang M, Lee Y, Song BR, Kang EH, Sim H, et al. Patient-derived cell-based pharmacogenomic assessment to unveil underlying resistance mechanisms and novel therapeutics for advanced lung cancer. J Exp Clin Cancer Res. 2023;42(1):37. 36717865 10.1186/s13046-023-02606-3 PMC9885631

[76] Zhou Q, Zhang XC, Tu HY, Gan B, Wang BC, Xu CR, et al. Biomarker-integrated study of single agent targeting molecular alterations of PI3KCA, MET, ALK, ROS1, KRAS, NRAS or BRAF in advanced NSCLC: phase 2 umbrella trial in China (CTONG1505). Ann Oncol. 2018;29:ix113.

[77] Juric D, Krop I, Ramanathan RK, Wilson TR, Ware JA, Sanabria Bohorquez SM, et al. Phase I dose-escalation study of Taselisib, an oral PI3K inhibitor, in patients with Advanced Solid tumors. Cancer Discov. 2017;7(7):704–15. 28331003 10.1158/2159-8290.CD-16-1080 PMC5501742

[78] Langer CJ, Redman MW, Wade JL 3rd, Aggarwal C, Bradley JD, Crawford J, Stella PJ, Knapp MH, Miao J, Minichiello K, Herbst RS, Kelly K, Gandara DR, Papadimitrakopoulou VA. SWOG S1400B (NCT02785913), a phase II study of GDC-0032 (Taselisib) for previously treated PI3K-Positive patients with stage IV squamous cell lung Cancer (Lung-MAP sub-study). J Thorac Oncol. 2019;14(10):1839–46. 31158500 10.1016/j.jtho.2019.05.029 PMC7017958

[79] Krop IE, Jegede OA, Grilley-Olson JE, Lauring JD, Mitchell EP, Zwiebel JA, et al. Phase II study of Taselisib in PIK3CA-Mutated Solid Tumors Other Than breast and squamous Lung Cancer: results from the NCI-MATCH ECOG-ACRIN Trial (EAY131) subprotocol I. JCO Precis Oncol. 2022;6:e2100424. 35138919 10.1200/PO.21.00424 PMC8865530

[80] Sato H, Yamamoto H, Sakaguchi M, Shien K, Tomida S, Shien T, et al. Combined inhibition of MEK and PI3K pathways overcomes acquired resistance to EGFR-TKIs in non-small cell lung cancer. Cancer Sci. 2018;109(10):3183–96. 30098066 10.1111/cas.13763 PMC6172047

[81] Juric D, de Bono JS, LoRusso PM, Nemunaitis J, Heath EI, Kwak EL, et al. A first-in-Human, phase I, dose-escalation study of TAK-117, a selective PI3Kα isoform inhibitor, in patients with Advanced Solid malignancies. Clin Cancer Res. 2017;23(17):5015–23. 28490463 10.1158/1078-0432.CCR-16-2888 PMC6858996

[82] Sanaei M-J, Razi S, Pourbagheri-Sigaroodi A, Bashash D. The PI3K/Akt/mTOR pathway in lung cancer; oncogenic alterations, therapeutic opportunities, challenges, and a glance at the application of nanoparticles. Translational Oncol. 2022;18:101364. 10.1016/j.tranon.2022.101364 PMC8850794 35168143

[83] Barlaam B, Cosulich S, Degorce S, Fitzek M, Green S, Hancox U, et al. Discovery of (R)-8-(1-(3,5-difluorophenylamino)ethyl)-N,N-dimethyl-2-morpholino-4-oxo-4H-chromene-6-carboxamide (AZD8186): a potent and selective inhibitor of PI3Kβ and PI3Kδ for the treatment of PTEN-deficient cancers. J Med Chem. 2015;58(2):943–62. 25514658 10.1021/jm501629p

[84] Choudhury AD, Higano CS, de Bono JS, Cook N, Rathkopf DE, Wisinski KB, et al. A phase I study investigating AZD8186, a potent and selective inhibitor of PI3Kβ/δ, in patients with Advanced Solid tumors. Clin Cancer Res. 2022;28(11):2257–69. 35247924 10.1158/1078-0432.CCR-21-3087 PMC9662946

[85] Siu LL, De Bono J, Wisinski KB, Higano CS, Cook N, De Miguel Luken MJ, et al. Abstract CT329: phase I study of the PI3Kβ/δ inhibitor AZD8186 in patients with advanced castration resistant prostate cancer, triple negative breast cancer, squamous non-small cell lung cancer or PTEN deficient solid tumors: update from dose-finding. Cancer Res. 2015;75(15Supplement):CT329–CT.

[86] Sun J, Zhang Z, Xia B, Yao T, Ge F, Yan F. Overexpression of PIK3CG in Cancer cells promotes Lung Cancer Cell Migration and Metastasis through enhanced MMPs expression and Neutrophil Recruitment and activation. Biochem Genet. 2024. 10.1007/s10528-024-10788-4 38602596

[87] Lauder SN, Smart K, Bart VMT, Pires A, Scott J, Milutinovic S, et al. Treg-driven tumour control by PI3Kδ inhibition limits myeloid-derived suppressor cell expansion. Br J Cancer. 2022;127(9):1595–602. 35986086 10.1038/s41416-022-01917-0 PMC9596434

[88] Burris HA, Flinn IW, Patel MR, Fenske TS, Deng C, Brander DM, et al. Umbralisib, a novel PI3Kδ and casein kinase-1ε inhibitor, in relapsed or refractory chronic lymphocytic leukaemia and lymphoma: an open-label, phase 1, dose-escalation, first-in-human study. Lancet Oncol. 2018;19(4):486–96. 29475723 10.1016/S1470-2045(18)30082-2

[89] Lukey PT, Harrison SA, Yang S, Man Y, Holman BF, Rashidnasab A, et al. A randomised, placebo-controlled study of omipalisib (PI3K/mTOR) in idiopathic pulmonary fibrosis. Eur Respir J. 2019;53(3):1801992. 30765508 10.1183/13993003.01992-2018

[90] Lonetti A, Cappellini A, Spartà AM, Chiarini F, Buontempo F, Evangelisti C, et al. PI3K pan-inhibition impairs more efficiently proliferation and survival of T-cell acute lymphoblastic leukemia cell lines when compared to isoform-selective PI3K inhibitors. Oncotarget. 2015;6(12):10399–414. 25871383 10.18632/oncotarget.3295 PMC4496363

[91] Hung T-H, Wu C-P, Chen S-F. Differential changes in Akt and AMPK Phosphorylation regulating mTOR activity in the Placentas of pregnancies complicated by fetal growth restriction and gestational diabetes Mellitus with large-for-gestational age infants. Front Med. 2021;8. 10.3389/fmed.2021.788969 PMC8685227 34938752

[92] Rahmani M, Aust MM, Attkisson E, Williams DC Jr., Ferreira-Gonzalez A, Grant S. Dual inhibition of Bcl-2 and Bcl-xL strikingly enhances PI3K inhibition-induced apoptosis in human myeloid leukemia cells through a GSK3- and bim-dependent mechanism. Cancer Res. 2013;73(4):1340–51. 23243017 10.1158/0008-5472.CAN-12-1365 PMC3578060

[93] Deng H, Xu Q, Li X-T, Huang X, Liu J-Y, Yan R, et al. Design, synthesis, and evaluation of antitumor activity in Pseudolaric acid B azole derivatives: Novel and potent angiogenesis inhibitor via regulation of the PI3K/AKT and MAPK mediated HIF-1/VEGF signaling pathway. Eur J Med Chem. 2024;278:116813. 39226705 10.1016/j.ejmech.2024.116813

[94] Zwang Y, Jonas O, Chen C, Rinne ML, Doench JG, Piccioni F et al. Synergistic interactions with PI3K inhibition that induce apoptosis. Elife. 2017;6. 10.7554/eLife.24523 PMC5479695 28561737

[95] Butler DE, Marlein C, Walker HF, Frame FM, Mann VM, Simms MS, et al. Inhibition of the PI3K/AKT/mTOR pathway activates autophagy and compensatory Ras/Raf/MEK/ERK signalling in prostate cancer. Oncotarget. 2017;8(34):56698–713. 28915623 10.18632/oncotarget.18082 PMC5593594

[96] Peng W, Zhang H, Yin M, Kong D, Kang L, Teng X, et al. Combined inhibition of PI3K and STAT3 signaling effectively inhibits bladder cancer growth. Oncogenesis. 2024;13(1):29. 39068158 10.1038/s41389-024-00529-y PMC11283499

[97] Nakanishi Y, Walter K, Spoerke JM, O’Brien C, Huw LY, Hampton GM, et al. Activating mutations in PIK3CB Confer Resistance to PI3K Inhibition and define a Novel Oncogenic Role for p110β. Cancer Res. 2016;76(5):1193–203. 26759240 10.1158/0008-5472.CAN-15-2201

[98] Browne IM, Okines AFC. Resistance to targeted inhibitors of the PI3K/AKT/mTOR pathway in advanced oestrogen-receptor-positive breast Cancer. Cancers. 2024;16(12):2259. 38927964 10.3390/cancers16122259 PMC11201395

[99] Bettazova N, Senavova J, Kupcova K, Sovilj D, Rajmonova A, Andera L, et al. Impact of PIK3CA gain and PTEN loss on mantle cell lymphoma biology and sensitivity to targeted therapies. Blood Adv. 2024;8(20):5279–89. 39158100 10.1182/bloodadvances.2024013205 PMC11497468

[100] Liao M, Yao D, Wu L, Luo C, Wang Z, Zhang J, et al. Targeting the Warburg effect: a revisited perspective from molecular mechanisms to traditional and innovative therapeutic strategies in cancer. Acta Pharm Sinica B. 2024;14(3):953–1008. 10.1016/j.apsb.2023.12.003 PMC10935242 38487001

[101] Lue HW, Podolak J, Kolahi K, Cheng L, Rao S, Garg D, et al. Metabolic reprogramming ensures cancer cell survival despite oncogenic signaling blockade. Genes Dev. 2017;31(20):2067–84. 29138276 10.1101/gad.305292.117 PMC5733498

[102] Hanker AB, Kaklamani V, Arteaga CL. Challenges for the Clinical Development of PI3K inhibitors: strategies to improve their impact in solid tumors. Cancer Discov. 2019;9(4):482–91. 30867161 10.1158/2159-8290.CD-18-1175 PMC6445714

[103] Scheffold A, Jebaraj BMC, Tausch E, Bloehdorn J, Ghia P, Yahiaoui A, et al. IGF1R as druggable target mediating PI3K-δ inhibitor resistance in a murine model of chronic lymphocytic leukemia. Blood. 2019;134(6):534–47. 31010847 10.1182/blood.2018881029 PMC8212352

[104] Ramón YCS, Sesé M, Capdevila C, Aasen T, De Mattos-Arruda L, Diaz-Cano SJ, et al. Clinical implications of intratumor heterogeneity: challenges and opportunities. J Mol Med (Berl). 2020;98(2):161–77. 31970428 10.1007/s00109-020-01874-2 PMC7007907

[105] Fruman DA, Chiu H, Hopkins BD, Bagrodia S, Cantley LC, Abraham RT. The PI3K pathway in Human Disease. Cell. 2017;170(4):605–35. 28802037 10.1016/j.cell.2017.07.029 PMC5726441

