# Supplementary material for: Targeting PI3K signaling in Lung Cancer: advances, challenges and therapeutic opportunities
Source: J Transl Med. 2025 Feb 14;23:184. doi: 10.1186/s12967-025-06144-8 (PMC11829425; doi:10.1186/s12967-025-06144-8)
Supplement: Supplementary file 1 — Supplementary Material 1 [file 12967_2025_6144_MOESM1_ESM.docx]

8 January 2025

Marcello Maggiolini, M.D.

Journal of Translational Medicine

Dear Professor Maggiolini,

**JTRM-D-24-15348**

Targeting PI3K Signaling in Lung Cancer: Advances, Challenges and Therapeutic Opportunities

Bitian Zhang; Ping-Chung Leung; William Chi-Shing Cho; Chun Kwok WONG; Dongjie Wang Journal of Translational Medicine

Thank you very much for your email dated 20 December 2024 in which you advised us that you would be willing to consider a revised version of the above manuscript.

Following the reviewers’ comments and questions, we have seriously revised our original manuscript. We are pleased in re-submitting our revised manuscript with tracked changes and our point-by-point responses to the reviewers’ comments via manuscript submission website.

Thank you for your kind attention.

Yours sincerely,

Chun Kwok WONG

Correspondence Author

Professor

Department of Chemical Pathology

The Chinese University of Hong Kong

Prince of Wales Hospital

Shatin, NT

Hong Kong

E.Mail: ck-wong@cuhk.edu.hk

Tel: (852) 3505 2964

Fax: (852) 2636 5090
