# Supplementary material for: Targeting PI3K signaling in Lung Cancer: advances, challenges and therapeutic opportunities
Source: J Transl Med. 2025 Feb 14;23:184. doi: 10.1186/s12967-025-06144-8 (PMC11829425; doi:10.1186/s12967-025-06144-8)
Supplement: Supplementary file 2 — Supplementary Material 2 [file 12967_2025_6144_MOESM2_ESM.docx]

Reviewer reports:

Reviewer #1: I read with great interest the manuscript titled "Targeting PI3K Signaling in Lung Cancer: Advances, Challenges, and Therapeutic Opportunities" by Zhang et al. While the manuscript is well-written and covers an interesting topic, it lacks significant novelty, as numerous reviews on this subject already exist. The authors are encouraged to revise their text by incorporating new and recent insights. Furthermore, although lung cancer is broadly categorized into non-small cell lung cancer (NSCLC) and small cell lung cancer (SCLC), the authors could enhance the manuscript by discussing PI3K signaling within specific histological subtypes. After reviewing the manuscript, the primary question that arises is: what new insights does this work offer?
Answer：We sincerely thank you for their insightful comments and constructive suggestions. In response to your feedback, we have made several enhancements and revisions in our manuscript.
This review comprehensively summarizes recent advances in PI3K research related to lung cancer, with a specific focus on PI3K-mediated immunity, PI3K-associated metabolic reprogramming, and the latest developments and challenges concerning PI3K inhibitors in lung cancer treatment. Unlike existing reviews, we offer an integrated perspective and provide new insights into the role of PI3K signaling in tumor progression and the application of PI3K inhibitors in lung cancer therapy.

While our previous manuscript covered the development of PI3K inhibitors in lung cancer therapy, we have now reinforced this section by incorporating the latest research progress. This includes detailed discussions on current clinical trials involving PI3K inhibitors and the underlying mechanisms contributing to resistance against these therapies (Lines 296–465). Furthermore, taking your recommendation into account, we have enhanced the manuscript by delineating the role of PI3K signaling within specific histological subtypes of lung cancer, namely lung adenocarcinoma (NSCLC), squamous cell lung cancer (NSCLC) as well as SCLC. This allows for a more nuanced understanding of PI3K’s function and its therapeutic implications across different lung cancer classifications. These revisions aim to address the concerns regarding novelty and specificity, thereby strengthening the contribution of manuscript to the field. We are grateful for your valuable suggestions, which have significantly improved the quality and depth of our review.

Thank you once again for your time and consideration.

Reviewer #2: Quite comprehensive article. Since the article is focussed on PI3K Signaling in Lung Cancer: Advances, Challenges and Therapeutic Opportunities, a tabular data compilation of existing therapies, targeted drugs available their limitations, and efficacy data would enhance the user understanding of current role / importance of targetting PI3K signalling in cancer.
Answer：Thank you for your insightful suggestion. In response, we have incorporated Table 1 into the revised manuscript, which provides a comprehensive compilation of existing therapies and targeted PI3K inhibitors currently undergoing clinical trials for lung cancer treatment. This table details the available drugs, their specific PI3K targets, and the corresponding clinical trial phases and numbers, thereby enhancing the reader’s understanding of the current role and significance of targeting PI3K signaling in cancer therapy. We believe this addition effectively addresses your recommendation and strengthens the overall quality of our article.

Reviewer #3: Title of the journal: Targeting PI3K Signaling in Lung Cancer: Advances, Challenges and Therapeutic Opportunities.

The journal current provides a landscape of targeting PI3K signaling pathway in lung cancer. This comprehensively highlights early-generation of non-selective compounds and their clinical efficacy, while discussing potential strategies against resistance mechanism.

1.      Title: The title is adequate.
Answer 1：Thank you for your positive feedback regarding the title.

2.      Abstract: The abstract is well defined.
Answer 2：We appreciate your affirmation of the abstract.

3.      Introduction: The introduction is compact. However, it is missing references in several sentences.
Answer 3：Thank you for your valuable suggestion. We have revised the introduction to include additional references (Lines 90– Line 118). We appreciate your insightful input.
4.      PI3K Inhibitors in Lung Cancer Therapy: This area needs major reconstruction. References are missing. A table listing the inhibitors and targeted pathway should be included.
Answer 4：Thank you for your constructive suggestions. We have summarized the clinical trial information on PI3K inhibitors relevant to lung cancer therapy in Table 1. This table provides a comprehensive overview of the available drugs for lung cancer therapy, their specific PI3K targets, and the corresponding clinical trial phases and identifiers. We believe that this revision enhances the readers' understanding of the current role and significance of targeting the PI3K signaling pathway in lung cancer therapy.

5.      Page 9, line 9: Where authors talk about 'PI3K inhibitors exert antitumor effects through multiple, interconnected mechanisms', a different subheading is recommended.
Answer 5：Thank you for your kind suggestion. We have added a new subheading titled “Mechanisms underlying PI3K inhibitors in lung cancer” (Line 402) to better organize this section.

6.      Page 9, line 49: 'PI3K inhibitors and development of resistance' could come under a separate subheading maybe? And a little bit well depicted.
Answer 6：Thank you for your helpful suggestion. We have introduced a new subheading titled “Mechanisms Underlying Resistance to PI3K Inhibitors” (Line 424) and have elaborated on this topic to provide a clearer depiction.
7.      'PI3K and Metabolic Control': In my perception, this should come before PI3K Inhibitors in Lung Cancer Therapy. The outline of the review should be as following: on the first part where authors talk about the PI3K signaling pathway in general, then PI3K signaling in lung cancer and finally targeting PI3K pathway in Lung Cancer Therapy.
Answer 7：Thank you for your valuable suggestion. We have reorganized the manuscript according to the proposed outline: beginning with a general overview of the PI3K signaling pathway, followed by its role in lung cancer, and culminating with strategies for targeting the PI3K pathway in lung cancer therapy. We believe this revision improves the logical flow of the review. Please refer to the revised manuscript for the changes.

8.      The figures need to be more comprehensive. Information is missing.
Answer 8：Thank you for your constructive feedback. We have revised Figure 1 to include additional details, making it more comprehensive. We hope the updated figure provides a clearer and more complete representation of the PI3K signaling pathway in the context of lung cancer therapy.

9.      Discussion: The discussion is concise.
Answer 9：Thank you for your positive feedback on the discussion section.

In conclusion, this review is comprehensive about understanding PI3K signaling in lung cancer. They address the existing research gaps between targeted investigations, and novel inhibitor development. Therefore, the manuscript can be considered for the publication after minor review.
